# Genome-Wide Analysis of Multidrug and Toxic Compound Extrusion (*MATE*) Family in *Gossypium raimondii* and *Gossypium arboreum* and Its Expression Analysis Under Salt, Cadmium, and Drought Stress

**DOI:** 10.1534/g3.118.200232

**Published:** 2018-05-24

**Authors:** Pu Lu, Richard Odongo Magwanga, Xinlei Guo, Joy Nyangasi Kirungu, Hejun Lu, Xiaoyan Cai, Zhongli Zhou, Yangyang Wei, Xingxing Wang, Zhenmei Zhang, Renhai Peng, Kunbo Wang, Fang Liu

**Affiliations:** * Research Base in Anyang Institute of Technology, State Key Laboratory of Cotton Biology/Institute of Cotton Research, Chinese Academy of Agricultural Science, Anyang 455000, Henan, China; † School of Physical and Biological Sciences, Jaramogi Oginga Odinga University of Science and Technology, 40601 Bondo, Kenya; ‡ Biological and Food Engineering, Anyang Institute of Technology, Anyang 455000, Henan, China

**Keywords:** *MATE* genes, *Gossypium arboreum*, *Gossypium raimondii*, phylogenetic tree analysis, GO annotation, cadmium, drought, stress, GenPred, Shared Data Resources, Genomic Selection

## Abstract

The extrusion of toxins and substances at a cellular level is a vital life process in plants under abiotic stress. The multidrug and toxic compound extrusion (*MATE*) gene family plays a large role in the exportation of toxins and other substrates. We carried out a genome-wide analysis of *MATE* gene families in *Gossypium raimondii* and *Gossypium arboreum* and assessed their expression levels under salt, cadmium and drought stresses. We identified 70 and 68 *MATE* genes in *G. raimondii* and *G. arboreum*, respectively. The majority of the genes were predicted to be localized within the plasma membrane, with some distributed in other cell parts. Based on phylogenetic analysis, the genes were subdivided into three subfamilies, designated as M1, M2 and M3. Closely related members shared similar gene structures, and thus were highly conserved in nature and have mainly evolved through purifying selection. The genes were distributed in all chromosomes. Twenty-nine gene duplication events were detected, with segmental being the dominant type. GO annotation revealed a link to salt, drought and cadmium stresses. The genes exhibited differential expression, with *GrMATE18*, *GrMATE34*, *GaMATE41* and *GaMATE51* significantly upregulated under drought, salt and cadmium stress, and these could possibly be the candidate genes. Our results provide the first data on the genome-wide and functional characterization of *MATE* genes in diploid cotton, and are important for breeders of more stress-tolerant cotton genotypes.

Plant production and yield quality are greatly affected by salt, drought and heavy metal pollution in most agricultural fields (Lutts and Lefèvre 2015). The reduction in crops production due to salt, drought and heavy metal pollution is estimated to be >50% compared with other stress factors (Tuteja 2010). Currently, it is estimated that >6% of agricultural land is affected by salinity (Munns 2005); similarly, the amount of precipitation has drastically declined and the available fresh water is not sufficient to meet the demands of both agricultural and domestic use (Tilman *et al.* 2002). Worldwide, cotton production is on the decline, mainly because of drought, salt and heavy metal toxicity such as cadmium (Cd) stress (Ellouzi *et al.* 2013; Yu *et al.* 2016).

Cotton plants have undergone physiological, biological and molecular modification to adjust to the ever-changing environmental and climate conditions that are compounded by heavy pollution of agricultural lands with heavy metals (Ali *et al.* 2013; Hu *et al.* 2013; Sarwar *et al.* 2017). The extrusion of toxins and substances at a cellular level is a vital life process of plant survival (Hill 2011). The group of genes involved in the exportation of toxins and other substrates are the multidrug and toxic compound extrusion (*MATE*) gene family (He *et al.* 2010). In the multidrug superfamily known as the oligosaccharidyl-lipid/polysaccharide exporter superfamily, only the *MATE* family is known to exhibit a functional mechanism as a secondary carrier. Secondary active transport is a form of active transport across a biological membrane in which a transporter protein couples the movement of an ion (typically Na^+^ or H^+^) down its electrochemical gradient to the uphill movement of another molecule or ion against a concentration/electrochemical gradient. Thus, energy stored in the electrochemical gradient of an ion is used to drive the transport of another solute against a concentration or electrochemical gradient (Hvorup *et al.* 2003).

In recent years, many *MATE* transcription factors have been reported in cotton, one of which is *GhTT12* (*Gossypium hirsutum*), found to be involved in the transportation of proanthocyanidins (Pas) from the cytoplasmic matrix to the vacuole (Gao *et al.* 2016). Although cotton is a moderate salt-tolerant crop, improving salt tolerance and enhancing drought resistance has become an urgent problem to be addressed in cotton breeding (Chinnusamy *et al.* 2005; Iqbal *et al.* 2011). Salt and drought stress tremendously reduces the yield quantity and quality in cotton (Dabbert and Gore 2014).

The *MATE* gene family has a wide distribution in both eukaryotes and prokaryotic organisms, and consists of multiple genes (Omote *et al.* 2006). The first two classes of *MATE* genes were obtained from *Vibrio parahaemolyticus* and *Escherichia coli*: *NorM* and *YdhE*, respectively (Morita *et al.* 1998). The MATE proteins mainly functions as transporter proteins, and are basically broadly categorized into four main families: the small multidrug resistance family, the resistance nodulation cell division family, the major facilitator superfamily, and the ATP-binding cassette superfamily (Paulsen *et al.* 1996). The *MATE* genes have been reported to enhance tolerance of a range of cation dyes, aminoglycosides, and fluoroquinolones, possibly through proton-motive force (Morita *et al.* 1998). In addition, several studies on the *MATE* gene family have shown that the MATE proteins are substrate-specific and facilitate the movement of defined substances within the plant (Zhao and Dixon 2009; Tiwari *et al.* 2014). In higher plants, the land plants that have lignified tissues (the xylem) for conducting water and minerals throughout the plant, the *MATE* genes have been found to be involved in the transportation and transiting of xenobiotic and other small organic molecules, such as inositol hexakisphosphate, yokonolide B, *p*-chlorophenoxyisobutyric acid, toyocamycin and terfestatin (Diener *et al.* 2001; Tiwari *et al.* 2014). Salt-responsive genes belonging to MATE efflux proteins have been reported to play a significant role in conferring salt tolerance in rice and chickpea (Nimmy *et al.* 2015). In addition, putative salt-responsive genes from the model plant *Arabidopsis thaliana* encoding the MATE efflux family have been identified and found to enhance salt tolerance (Li *et al.* 2002). Drought affects crop productivity worldwide, and under drought conditions, the abscisic acid (ABA) level in plants increases sharply, resulting in stomatal closure and induction of stress genes (Nakashima *et al.* 2014). Therefore, ABA is believed to be a key player in drought stress response (Zhang *et al.* 2006). In *A. thaliana*, the DTX/MATE family member *AtDTX50* functions as an ABA efflux transporter, thus enhancing drought tolerance in plants (Zhang *et al.* 2014). Cd-regulated transporter genes, such as MATE family transporters and PDR, have been reported to be highly upregulated in the root tissues of *Oryza sativa* when exposed to Cd stress, suggesting a role for *MATE* and *PDR* genes in Cd detoxification via export of Cd from the cytoplasm (Ogawa *et al.* 2009).

A number of genome-wide studies and expression analyses of *MATE* genes have been conducted in soya bean (Liu *et al.* 2016), blueberry (Chen *et al.* 2015), *Zea mays* (Zhu *et al.* 2016), and other plants, but no work has been reported on diploid cotton to date, despite multiple studies on *MATE* gene families. Cotton is considered to be the foremost important natural fiber crop and is the textile industry’s most indispensable raw material globally (Chakravarthy *et al.* 2012; Zhou *et al.* 2014). Cotton is currently grown in many countries worldwide, and is a major cash crop for foreign exchange (Chakravarthy *et al.* 2014). The complete sequencing of the two diploid cotton genomes, *Gossypium raimondii* (D genome) and *Gossypium arboreum* (A genome) (Wang *et al.* 2012; Li *et al.* 2014), has provided the valuable resources for the study of cotton at the gene level.

Given the potential roles of MATE proteins in the regulation of gene expression in response to abiotic stresses, it is of the utmost interest to carry out a genome-wide survey of this gene family in the two diploid parental lines of upland cotton, *G. raimondii* (D genome) and *G. arboreum* (A genome). In this work, we identified 70 and 68 *MATE* genes in *G. raimondii* and *G. arboreum*, respectively, analyzed their phylogenetic tree relationships, chromosomal positions, duplicated gene events, gene structure, and performed a profiling analysis of gene expression on cotton root tissue. Our findings provide the very first foundation and detailed analysis of the role of *MATE* genes in salt, Cd and drought stress response, and shows how cotton seedlings adapt root phenology in response to the overall effect of the stresses.

## Materials and Methods

### Identification of MATE genes family

The conserved domain of MATE protein was downloaded using a hidden Markov model (HMM) (PF01554). To identify the MATE proteins in cotton, the HHM profile of MATE protein was subsequently used as a query in an HMMER search (http://hmmer.janelia.org/) (Finn *et al.* 2011) against the genome sequences of *G. raimondii* and *G. arboreum*. The genome sequence of *G. arboreum* was obtained from the Cotton Genome Project (http://www.cgp.genomics.org.cn) and *G. raimondii* and *A. thaliana* genomes were downloaded from Phytozome (http://www.phytozome.net/), with *E*-value <0.01. All the redundant sequences were discarded from further analysis based on cluster W^17^ alignment results. Furthermore, SMART and PFAM databases were used to verify the presence of the *MATE* gene domains. The isoelectric points and molecular weights of MATE proteins were estimated with the ExPASy Server tool (http://web.expasy.org/compute_pi/). In addition, subcellular location prediction of GrMATE and GaMATE proteins was determined with online tools TargetP1.1 (http://www.cbs.dtu.dk/services/TargetP/) server (Emanuelsson *et al.* 2007) and Protein Prowler Subcellular Localization Predictor version 1.2 (http://bioinf.scmb.uq.edu.au/Pprowler_webapp_1-2/) (Bodén and Hawkins 2005). Validation and determination of the possible cell compartmentalization, as obtained by the two software programs, was done by WoLF PSORT (https://wolfpsort.hgc.jp/) (Horton *et al.* 2007).

### Chromosomal locations, gene duplication and syntenic analysis

The chromosomal distribution of the *MATE* genes were mapped on the cotton chromosomes based on gene position by mapchart 2.2 software (Voorrips 2002). We performed a syntenic analysis of the diploid cottons in relation to the distribution of the *MATE* genes in their respective genome, and drew a pictorial diagram with the online tool Circos-0.69 (http://circos.ca/) (Krzywinski *et al.* 2009). Homologous genes of *G. raimondii* and *G. arboreum* were identified by BLASTP, with threshold >80% similarity and at least 80% alignment ratio to their protein total lengths. Default parameters were maintained in all steps. The synonymous substitution (ds) and nonsynonymous substitution rates (dn) for the paralogous gene pairs were estimated by SNAP (https://www.hiv.lanl.gov/content/sequence/). Tandem duplications were designated as multiple genes of one family located within the same or neighboring intergenic region (Du *et al.* 2013).

### Phylogenetic analyses and gene structure organization of the MATE proteins in cotton

Full-length sequences of *G. arboreum*, *G. raimondii*, and *A. thaliana* MATE proteins were first aligned using ClustalW (Larkin *et al.* 2007). We then used MEGA 6 to conduct phylogenetic analyses based on protein sequences, using the neighbor-joining (NJ) method (Tamura *et al.* 2013). Support for each node was tested with 1000 bootstrap replicates. The gene structures were obtained by comparing the genomic sequences and their predicted coding with an online gene structure displayer server (http://gsds.cbi.pku.edu.cn/), as previously used for the analysis of *LEA* genes in cotton (Magwanga *et al.* 2018).

### Promoter cis-element analysis

Promoter sequences (1 kb up and down stream of the translation start site) of all *MATE* genes were obtained from the Cotton Genome Project. Transcriptional response elements of *GaMATE* and *GrMATE* gene promoters were predicted using the PLACE database (http://www.dna.affrc.go.jp/PLACE/signalscan.html) (Higo *et al.* 1999).

### Gene ontology annotation

The functional grouping of the MATE proteins’ sequences and the analysis of their annotation data were executed using Blast2GO PRO software version 4.1.1 (https://www.blast2go.com). Blast2GO annotation associates genes or transcripts with gene ontology (GO) terms, using hierarchical terms. Genes were described using three categories of GO classification: molecular function (MF), biological processes (BP), and cellular components (CC).

### Tertiary protein structure prediction

The protein sequences of MATEs were analyzed by Phyre2, a protein-modeling server (http://www.sbg.bio.ic.ac.uk/*phyre2). The results were obtained in the form of protein database files, which were then submitted to PoreWalker server to predict their individual tertiary protein structures in relation to pore size (http://www.ebi.ac.uk/thornton-srv/software/PoreWalker/). To validate the secondary structural information, we performed further analysis by submitting the protein sequences of the *MATE* genes to an online tool, Protter (http://wlab.ethz.ch/protter/), for visualization of proteoforms and interactive integration of annotated and predicted sequence features together with their experimental proteomic evidence.

### Plant materials and treatment

Healthy seeds from species *G. raimondii* and *G. arboreum* were delinted and pretreated; *G. raimondii* seeds have hard seed testa, thus a small slit was made before germinating the seeds. The seeds were germinated on wet filter paper for 3 d at 25°. The seedlings were then transferred to a hydroponic setup with Hoagland nutrient solution (Hoagland and Arnon 1950), in the greenhouse with conditions set at 28° day/25° night, 14 hr photoperiod, and 60–70% relative humidity. The cotton seedlings at three-true-leaves stage were subjected to stress, by transferring to a nutrient solution with 250 mM sodium chloride (NaCl), 500 µM cadmium chloride (CdCl_2_), or 15% PEG-6000, for salt, heavy metal, and drought stress, respectively. Root tissues were the main target organ system; roots were then collected for RNA extractions at 0, 3, 6, 12 and 24 hr posttreatment. Untreated plants served as the control. Each treatment had three replications. For each biological replicate, the roots were collected from two individual seedlings to ensure that a sufficient amount of RNA was extracted for qRT-PCR analysis per treatment. The root samples were immediately frozen in liquid nitrogen on collection, and stored at −80° until RNA extraction.

### RNA isolation and qRT-PCR verification

We used an RNA extraction kit, EASYspin plus plant RNA kit (Aid Lab, Biotech, Beijing, China), for the RNA extraction. The quality and concentration of each RNA sample were determined by gel electrophoresis and a NanoDrop 2000 spectrophotometer, and only RNA that met the criterion 260/280 for 1.8–2.1 or 260/230 for ≥2.0 were used for further analyses. The cotton constitutive *Ghactin7* gene (forward sequence 5′ATCCTCCGTCTTGACCTTG3′; reverse sequence 5′TGTCCGTCAGGCAAC TCAT3′) was used as a reference gene and specific *MATE* gene primers were applied for qRT-PCR analysis. The first-strand cDNA synthesis was carried out with TranScript-All-in-One First-Strand cDNA Synthesis SuperMix (TransGen Biotech, Beijing, China) for qPCR, in accordance with the manufacturer’s instructions. Primer Premier 5 was used to design 87 MATE primers (Supplemental Material, Table S1), with melting temperatures of 55–60°, primer lengths of 18–25 bp, and amplicon lengths of 101–221 bp. Details of the primers are shown in Table S1. Fast Start Universal SYBRgreen Master (Rox) (Roche, Mannheim, Germany) was used to perform qRT-PCR in accordance with the manufacturer’s instructions. Reactions were prepared in a total volume of 20 μl, comprising 10 μl of SYBR green master mix, 2 μl of cDNA template, 6 μl of ddH_2_O, and 2 μl of each primer for a final concentration of 10 μM. The *Ghactin7* was used as a reference gene. The PCR thermal cycling conditions were as follows: 95° for 10 min, 40 cycles of 95° for 5 sec, 60° for 30 sec, and 72° for 30 sec. Data were collected during the extension step: 95° for 15 sec, 60° for 1 min, 95° for 30 sec, and 60° for 15 sec. Three biological replicates and three technical replicates were performed per cDNA sample.

### Data availability

The authors state that all data necessary for confirming the conclusions presented in the article are represented fully within the article. Supplemental material available at Figshare: https://doi.org/10.25387/g3.5970889.

## Results

### Identification of MATE genes in cotton

The HMM profile of the Pfam MATE domain (PF01554) was used as the query to identify the *MATE* genes from the two diploid cotton A and D genomes. Seventy three (73) and 72 *MATE* genes were identified in *G. raimondii* and *G. arboreum*, respectively. All of the *MATE* genes were analyzed manually, using SMART and PFAM databases to verify the presence of the *MATE* gene domain. Finally, 68 and 70 candidate *MATE* genes were identified in *G. arboreum* and *G. raimondii*, respectively. All the identified *MATE* genes were designated as GaMATE 1–68 for *G. arboreum* and GrMATE1–70 for *G. raimondii* (Table 1). The MATE protein domains were further analyzed for their conserved domain, using the conserved domain database (CDD) tool hosted by NCBI (Table S2). Protein domain analysis revealed a minimum of 3 to a maximum of 12 signature transmembrane domains (TMs) in all the MATE proteins in the two diploid cotton, indicating that all of the MATE proteins were members of membrane proteins (Table S3). The proteins encoding the *MATE* genes were varied in length, with GrMATE protein lengths ranging from 229 to 601 aa and predicted molecular weights ranging from 24.78 to 66.28 kDa, and GaMATE protein lengths ranging from 153 to 722 aa and predicted molecular weights ranging from 16.72 to 78.90 kDa (Table S4). In relation to amino acid length proportions, 92.86% of GrMATE proteins and 94.12% of GaMATE proteins consisted of 441–554 and 435–570 aa, respectively. In addition, the majority of the proteins were found to possess 10–12 TMs, which suggested that the MATE protein lengths were highly conserved in the two cotton genomes. The results obtained for GrMATE and GaMATE are consistent with previous findings in which the MATE transporter proteins have been found to possess more or less than 12 TMs in some species (Li *et al.* 2002), 14 TMs in the FRD3 protein (Green 2004), and 9–11 TMs in EDS5 (Nawrath 2002).

**Table 1:** Classification of the *MATE* gene family and distribution across the chromosomes of *G. arboreum* and *G. raimondii*

Cotton Genome	Chromosome	Subfamilies			Total
		M1	M2	M3	
*Gossypium arboreum* (AA)	A1	4	2	0	6
	A2	1	0	1	2
	A3	5	0	0	5
	A4	3	3	0	6
	A5	3	1	0	4
	A6	4	2	0	6
	A7	2	2	2	6
	A8	2	2	0	4
	A9	5	1	0	6
	A10	7	5	1	13
	A11	1	2	0	3
	A12	0	0	2	2
	A13	0	0	3	3
	Scaffold	2	0	0	2
	Subtotal	39	20	9	68
	Percentage (%)	57.35	29.42	13.24	100
*Gossypium raimondii* (DD)	D1	2	1	0	3
	D2	3	0	2	5
	D3	0	2	0	2
	D4	2	2	2	6
	D5	5	1	3	9
	D6	2	3	2	7
	D7	6	2	0	8
	D8	4	1	0	5
	D9	8	3	1	12
	D10	1	2	0	3
	D11	2	2	0	4
	D12	0	0	1	1
	D13	5	0	0	5
	Subtotal	40	19	11	70
	Percentage (%)	57.14	27.14	15.72	100

A, A genome of *G. arboreum*; D, D genome of *G. raimondii*.

The pI values of the predicted proteins were varied in both of the cotton genomes: in *G. raimondii*, the pI values ranged from 4.59 to 9.5 (for example, GrMATE39 had a pI value of 4.59, whereas GrMATE65 had a pI value of 9.5), and in *G. arboreum*, the pI values ranged from 5 to 9.53 (the lowest pI value was obtained for GaMATE10, with a pI value of 5, whereas GaMATE8 had the highest pI value of 9.53). The results were in agreement with previous reports on the identification and expression analysis of *MATE* genes in blueberry plants (Chen *et al.* 2015). WoLF PSORT was used to predict the subcellular location of the various MATE proteins. The results obtained by WoLF PSORT were further validated by reanalyzing the various protein sequences with the TargetP1.1 (http://www.cbs.dtu.dk/services/TargetP/) server (Emanuelsson *et al.* 2007) and Protein Prowler Subcellular Localization Predictor version 1.2 (http://bioinf.scmb.uq.edu.au/pprowler_webapp_1-2/) (Bodén and Hawkins 2005). The results obtained for the three methods were consistent, with half of the entire GaMATE proteins found to be involved in secretory pathways, and the same observed for GrMATEs. The high number of the MATE proteins involved in secretory pathways gives a stronger indication of the vital role played by these proteins in the translocation, folding, cargo transport, and exocytosis of various secretory products, including toxins from the cell. For the subcellular localization prediction for the *GrMATE* genes, eight genes were found to be chloroplast proteins, five genes were cytoplasmic proteins, a single gene each was located in the extracellular structures and mitochondrion, four genes were vacuolar proteins, and the largest proportions of *GrMATE* genes were found to be compartmentalized within the plasma membrane (51 genes), accounting for over 72% of all GrMATEs detected in *G. raimondii*.

The subcellular predictions of the MATE proteins from *G. arboreum* (GaMATEs), were more or less similar to the predicted localization of the MATE proteins in *G. raimondii*: six different cell structures were found to harbor the *GaMATE* genes, in which the highest proportion was detected in the plasma membrane (54 genes), accounting for >75% of all GaMATE genes found in *G. arboreum*. In other cell structures and organelles, they were low in distribution: four genes were found in chloroplasts, two genes were found in the cytoplasm, and six genes were found in the vacuoles, with a single gene each found in the endoplasmic reticulum and the nucleus. The high proportions of MATE proteins were predicted to be localized within the plasma membrane, and the results obtained are consistent with previous findings, in which 82.91% (97 out of 117 MATE proteins) of the MATE transporter proteins in *Glycine max* were found to be located in the plasma membrane (Liu *et al.* 2016). The detection of proteins encoding *MATE* genes being localized within the plasma membrane explains their primary role of maintaining membrane integrity through the exclusion of toxins from the plants. The subcellular localization, gene identity, molecular weight, and other gene descriptions are illustrated in Table S4.

### Phylogenetic analyses of the MATE proteins in cotton with A. thaliana

**Figure 1:**
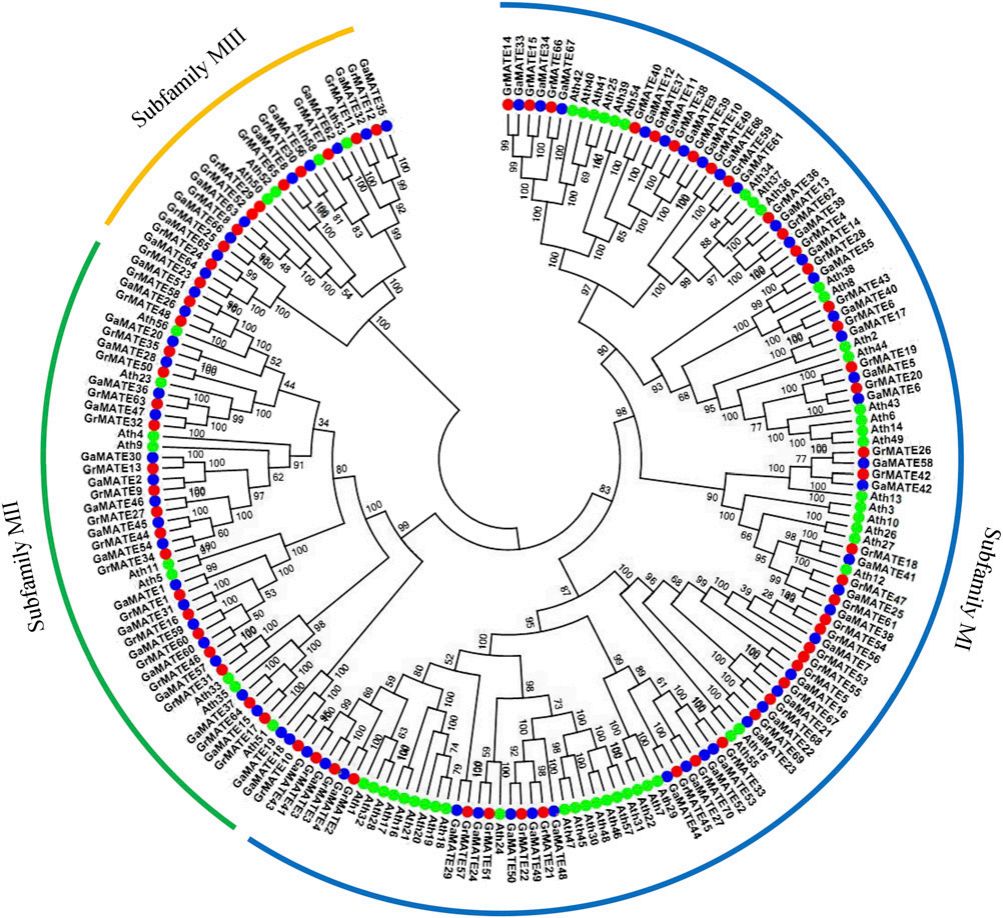
Phylogenetic relationship of *MATE* genes in two diploid cotton species with *Arabidopsis*. Neighbor-joining phylogeny of 68 genes for *G. arboreum*, 70 genes for *G. raimondii*, and 58 *Arabidopsis* MATE protein sequences, as constructed by MEGA 6.0. The different colors mark the various *MATE* gene types.

In order to understand the evolutionary history and relationships of *MATE* gene family in cotton in relation to other plants, multiple sequence alignment of 68 genes for *G. arboreum*, 70 genes for *G. raimondii*, and 58 *Arabidopsis* MATE protein sequences were analyzed. The bootstrap values for some nodes of the NJ tree were low due to long sequence similarities; confirmation was done by the NJ method and by reconstructing the phylogenetic tree with the minimal evolution method. The trees produced by the two methods were identical, suggesting that the two methods were consistent. Based on the phylogenetic tree analysis, the *MATE* genes in cotton were classified into three subfamilies, designated as M1, M2 and M3. Subfamily M1 was the largest group, with 124 genes accounting for 63% of the entire proteins encoding the *MATE* genes, in which 40 (57%) were from *G. raimondii*, 39 (57%) were from *G. arboreum* and 45 (78%) were from *Arabidopsis*. The second largest subfamily was M2 ,with 48 (24%) of the proteins encoding the *MATE* genes, with 20, 19, and 9 genes from *G. arboreum*, *G. raimondii*, and *A. thaliana*, respectively. The smallest subfamily was M3, with 9, 11, and 4 *MATE* genes in *G. arboreum*, *G. raimondii*, and *A. thaliana*, respectively (Figure 1). Classifications of the MATE proteins varied from plant to plant; for instance, in soya beans, four subfamilies were identified (Liu *et al.* 2016), and in maize, seven groups have been reported for MATE proteins (Zhu *et al.* 2016), and therefore the classification adopted in this study was accurate and conforms to previous findings.

**Figure 2:**
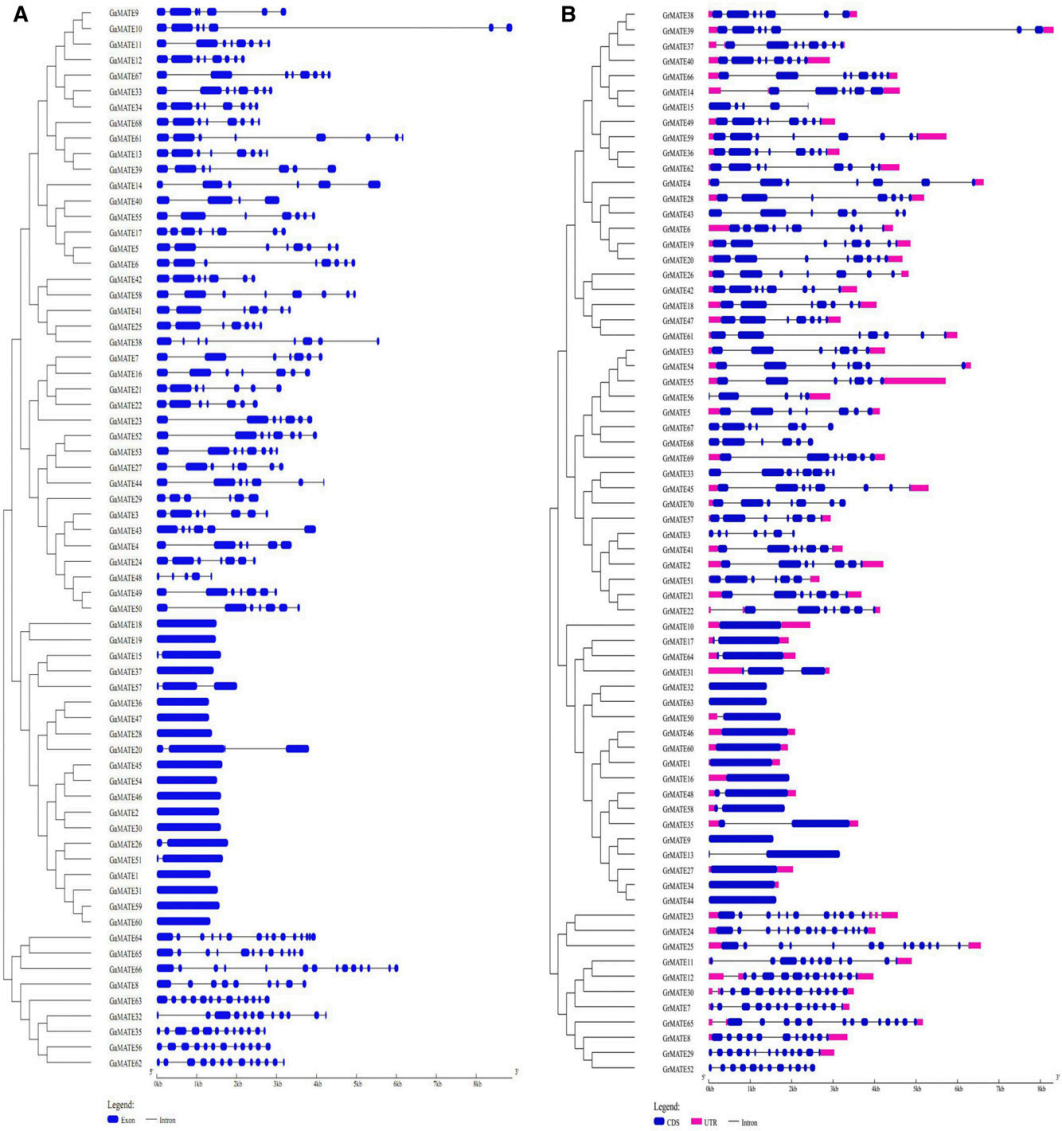
Phylogenetic tree and gene structure of *MATE* genes in diploid cotton. The phylogenetic tree was constructed using MEGA 6.0. Exon/intron structures of *MATE* genes are shown. A. Phylogenetic tree and structure for *MATE* genes of *G. arboreum*. B. Phylogenetic tree and structure for *MATE* genes of *G. raimondii*.

Gene structural diversity and conserved motif divergence are possible mechanisms for the evolution of multigene families (Hu *et al.* 2010). In order to gain further information on the structural diversity of cotton *MATE* genes, we analyzed the exon-intron organization in full-length cDNAs with corresponding genomic DNA sequences for each *MATE* gene in cotton (Figure 2). Most closely related *MATE* gene members within the same group shared similar gene structures in terms of either intron numbers or exon lengths. For example, for the *MATE* genes in the subfamily M3 in G. *arboreum* and *G. raimondii*, all gene structures were disrupted by the highest number of introns, with 8–14 introns disruption. The second largest, in terms of intron disruption, were members of the subfamily M1, with three to eight introns disruption. A unique observation was made among the members of the subfamily M2: all genes had the least intron disruptions, in which some were found to be intronless in both *GrMATE* and *GaMATE* genes, with those disrupted having one to three introns. The results were in agreement with previous studies that reported *MATE* genes located from different subfamilies to be generally distinct, with each group sharing a common gene structural layout (Zhu *et al.* 2016).

The clustering analysis showed three main subfamilies, which were designated as subfamily M1, M2 and M3 (Figure 2). In the subfamily M1, *GrMATE2*, *GaMATE4*, *GrMATE3*, *GaMATE3*, *GrMATE41* and *GaMATE43* were clustered together with a *MATE*-type gene *AtDTX1* (AT2G04070). Annotated as *Ath19* in the phylogenetic tree, *AtDTX1* is known to function as an efflux carrier for plant-derived alkaloids, antibiotics, and other toxic compounds. Interestingly, *AtDTX1* also has the ability of detoxifying Cd^2+^, and is known as a heavy metal flavonoid transporter (Li *et al.* 2002). Furthermore, experimental results suggest that *AtDTX1* is localized in the plasma membrane in plant cells, thereby mediating the efflux of plant-derived or exogenous toxic compounds from the cytoplasm (Li *et al.* 2002). *AtTT12* is homologous to *Ath13* but orthologous to *GrMATE26*, *GaMATE58*, *GrMATE42* and *GaMATE42*. *AtTT12* was presumed to be a vacuolar transporter for flavonoids in the seed coat, but later found to be expressed specifically in cells synthesizing Pas (Marinova *et al.* 2007). *AtTT12* is orthologous to a number of GrMATEs and GaMATEs in subfamily M1, and has diverse potential functions such as transport and accumulation of flavonoids or alkaloids, extrusion of plant-derived or xenobiotic compounds, regulation of disease resistance, and response to abiotic stresses (Liu *et al.* 2016). It provides stronger evidence for the significant role played by the cotton *MATE* genes in enhancing tolerances to various abiotic stress factors. It has been found that flavonoid concentrations increase with an increase in drought stress (Lama *et al.* 2016), which implies that the *GrMATE* and *GaMATE* genes do play a significant role in enhancing drought tolerance in cotton. *GrMATE26*, *GaMATE58*, *GrMATE42* and *GaMATE42* are functional orthologous genes to *AtTT12*, and all could be involved in the transportation of epicatechin 3′-*O*-glucoside with higher affinity and velocity than cyanidin 3-*O*-glucoside (Zhao *et al.* 2011). It has been found that *DTX35*, a subtype of the *MATE* gene type known as tonoplast detoxification efflux carrier (DTX), is homologous to *Ath8* and *Ath38*, which are orthologous to *GrMATE28*, *GaMATE55*, *GrMATE43* and *GaMATE40*, and function as chloride channels, which is highly significant for the regulation of turgidity and reduction of salt toxicity in *Arabidopsis* (Zhang *et al.* 2017).

The presence of pore-forming amino acids in MATE proteins enhances their substrate specificity, and similar attributes have been found among aquaporins, which are known to be substrate-specific due to their hydrophobicity and the size of their pore-forming amino acids (Lee *et al.* 2005; Törnroth-Horsefield *et al.* 2006). The chloride channel plays a role in sequestration of anions, including nitrate and chloride, into the vacuole, thus reducing the danger of salt toxicity within the plant cell (Zifarelli and Pusch 2010). The fact that all of the *MATE* genes obtained from *Arabidopsis* were clustered together with either the *GrMATE* and or *GaMATE* genes provides an indication that these genes could play a vital role in enhancing drought tolerance in diploid cotton. The *MATE* gene type *AtDTX1*, a *MATE* gene from *Arabidopsis*, is known for its relatively broad substrate specificity and confers Cd tolerance when expressed in *E. coli* (Li *et al.* 2002). Thus, we conclude that *GrMATE* and *GaMATE* genes may be involved in salt, drought and Cd stress-tolerance enhancement in diploid cotton.

### Chromosomal distribution of cotton genes encoding MATE proteins transporters

**Figure 3:**
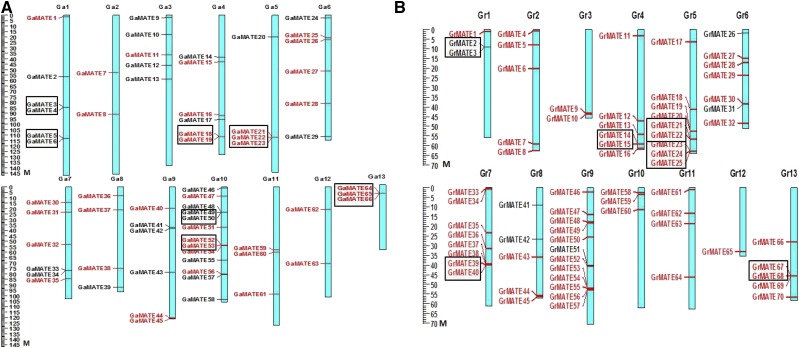
*MATE* gene distribution in genome A and genome D cotton chromosomes. A: chromosome mapping of *GaMATE* genes; B: chromosome mapping of *GrMATE* genes. Chromosomal position of each *MATE* gene was mapped according to the diploid cotton genome. The chromosome number is indicated at the top of each chromosome. Red indicates genes that showed a high level of collinearity. Duplicated genes are shown in black boxes.

To unearth the chromosomal locations of cotton *MATE* genes based on their positions, data retrieved from the whole cotton genome sequences were used. Chromosome distribution was done by BLASTN search against *G. arboreum* from the Cotton Genome Project, and *G. raimondii* genome database in Phytozome (http://www.phytozome.net/cotton.php). Seventy *G. raimondii MATE* genes (GrMATEs) were all mapped by mapchart, whereas only 66 genes of *G. arboreum* were mapped, two of which were scaffold. A plot of *MATE* genes on the cotton genome showed that the MATE loci are found on every chromosome, which is in agreement with previous results for the mapping of *MATE* genes in *Z. mays*, and the *MATE* genes were distributed across all 10 chromosomes (Zhu *et al.* 2016). The distribution of the mapped *MATE* genes in both of the two diploid genomes was asymmetrical in nature. In genome A (*G. arboreum*), a high density of these loci was observed on chromosome 10, with 13 genes, translating to 19% of all *GaMATE* genes in genome A, whereas the lowest loci density was observed for chromosome 12, with only two *GaMATE* genes, which accounted for only 3% of all the *GaMATE* genes. The mapping of the gene loci were not uniform in genome D (*G. raimondii*): the highest loci density was noted in chromosome 9, with 12 genes, which translated to 17% of the genes, and the lowest loci density was in chromosome 12, with only a single gene (Figure 3). In the distribution of the *MATE* genes in the two diploid cotton genomes, there was variation in relation to the number of *MATE* genes; for example, chromosome 10 in *G. raimondii* had only three *GrMATE* genes compared to its homolog chromosome in *G. arboreum* with 13 *GaMATE* genes. The wider distribution of the *MATE* genes could possibly explain their roles within the plant cell. In this study, the genes were found to have uneven distribution in all of the 13 cotton chromosomes. Our results are consistent with previous reports on the distribution and chromosome patterning of the *MATE* genes in soya beans and maize (Liu *et al.* 2016; Zhu *et al.* 2016). The difference in gene loci could possibly be due to gene duplication, gene loss, and/or chromosomal rearrangement as evident on the *LEA* gene distributions in the two diploid cotton chromosomes (Magwanga *et al.* 2018).

### Gene duplication and syntenic analysis

**Figure 4:**
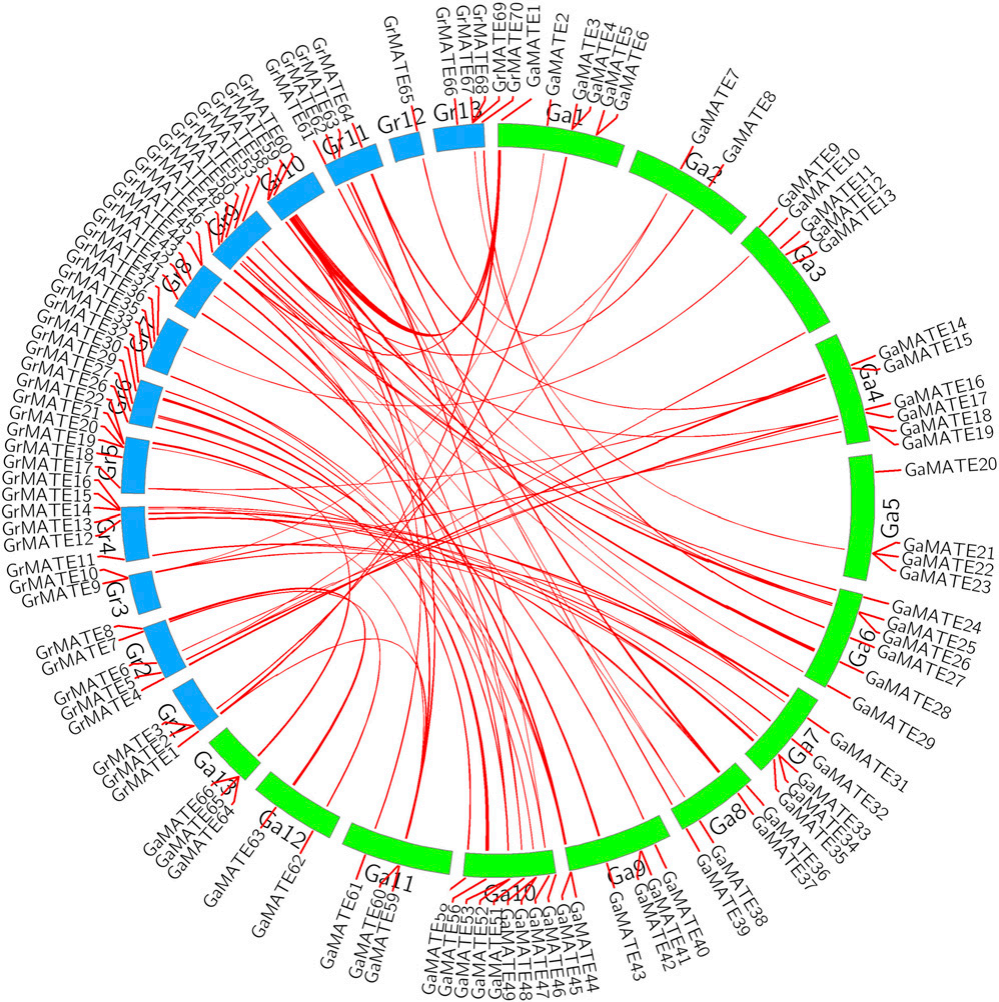
Syntenic relationships among *MATE* genes from *G. raimondii* and *G. arboreum. G. raimondii* and *G. arboreum* chromosomes are indicated in different colors. The putative orthologous *MATE* genes between *G. raimondii* and *G. arboreum* are represented in red. The chromosome number and the gene names are indicated around the outside of the figure.

Duplicated genes have been found to play a role in stress response, development, signaling, and transcriptional regulation, which are needed for the extension and formation of gene families that are found across different genomes (Innan and Kondrashov 2010). To analyze the relationships between *MATE* genes and gene duplication events, we combined syntenic blocks of *MATE* genes in *G. raimondii* and *G. arboreum* (Figure 4). The ds/dn ratios for all the paralogous gene pairs were <1, which indicated that the cotton *MATE* genes have undergone purifying selection and their structures are highly conserved in nature (Table 2). A total of 29 *MATE* genes were duplicated across the two cotton genomes, with the most duplicated genes detected in genome A (16 genes, translating to ∼55% of all the duplicated genes, whereas in *G. raimondii* there were only 13 gene duplication events, accounting for only 45% of duplicated genes). A single type of gene duplication event was detected, namely the segmental type. In a syntenic analysis, 43 *GaMATE* and 45 *GrMATE* genes were found to have undergone segmental duplication, in which the proportion of *GaMATE* genes accounted for 63.2% whereas the *GrMATE* genes accounted for 64.3%; this clearly indicates that the major duplication type in the evolution of the diploid cotton *MATE* genes was segmental. Segmental gene duplication has been proven to be a major contributing factor during the evolution time of various genes, for instance, in myeloblastosis (MYBs) (Salih *et al.* 2016) and *LEA* genes (Magwanga *et al.* 2018). In the analysis of duplication events on maize *MATE* genes, more genes were found to have evolved through segmental as opposed to tandem duplication (Zhu *et al.* 2016). The syntenic analysis results further showed the level of segmental duplication, as illustrated in (Figure 4).

**Table 2:** Estimation of synonymous (ds) and nonsynonymous (dn) substitution rate for the paralogous *MATE* genes in cotton

Paralogous Gene Pairs		SD	Sn	S	N	ps	pn	ds	dn	ds/dn	ps/pn	Purifying Selection
GaMATE9	GaMATE27	218.1667	817.8333	322.5	1090.5	0.6765	0.75	1.7419	7.4136	0.235	0.902	Yes
GrMATE3	GrMATE17	112.3333	394.6667	163.6667	526.3333	0.6864	0.7498	1.8501	6.3474	0.2915	0.9153	Yes
GaMATE20	GaMATE35	265.8333	907.1667	380.1667	1209.833	0.6993	0.7498	2.0199	6.2844	0.3214	0.9326	Yes
GaMATE29	GaMATE63	212.3333	689.6667	298.3333	919.6667	0.7117	0.7499	2.2316	6.7659	0.3298	0.9491	Yes
GrMATE46	GrMATE48	278.8333	918.1667	389.6667	1224.333	0.7156	0.7499	2.3108	6.9805	0.331	0.9542	Yes
GaMATE37	GrMATE3	112.3333	391.6667	167.1667	522.8333	0.672	0.7491	1.6974	5.0638	0.3352	0.897	Yes
GaMATE67	GrMATE5	238.8333	841.1667	333.3333	1121.667	0.7165	0.7499	2.3314	6.9148	0.3372	0.9554	Yes
GrMATE21	GrMATE40	243.3333	839.6667	344.1667	1119.833	0.707	0.7498	2.1445	6.2264	0.3444	0.9429	Yes
GrMATE8	GrMATE44	233.3333	834.6667	354.5	1115.5	0.6582	0.7482	1.5754	4.543	0.3468	0.8797	Yes
GaMATE44	GaMATE64	223.5	768.5	313.1667	1024.833	0.7137	0.7499	2.2707	6.543	0.347	0.9517	Yes
GrMATE34	GrMATE64	268.6667	870.3333	369.5	1160.5	0.7271	0.75	2.617	7.4602	0.3508	0.9695	Yes
GaMATE28	GrMATE44	221.8333	795.1667	324.6667	1061.333	0.6833	0.7492	1.8145	5.1464	0.3526	0.912	Yes
GrMATE44	GrMATE50	224.1667	793.8333	326.5	1059.5	0.6866	0.7493	1.8527	5.1836	0.3574	0.9163	Yes
GaMATE62	GaMATE63	260.3333	865.6667	372.1667	1154.833	0.6995	0.7496	2.0237	5.6581	0.3577	0.9332	Yes
GaMATE27	GrMATE52	234.3333	796.6667	340.6667	1063.333	0.6879	0.7492	1.8681	5.1479	0.3629	0.9181	Yes
GaMATE45	GrMATE8	236.1667	834.8333	354.1667	1115.833	0.6668	0.7482	1.6493	4.5119	0.3655	0.8913	Yes
GrMATE12	GrMATE53	241.5	807.5	339	1077	0.7124	0.7498	2.2446	6.0604	0.3704	0.9501	Yes
GaMATE7	GrMATE54	215.8333	806.1667	330.6667	1079.333	0.6527	0.7469	1.5319	4.1193	0.3719	0.8739	Yes
GaMATE63	GrMATE29	213	748	324.8333	1001.167	0.6557	0.7471	1.5554	4.1739	0.3726	0.8777	Yes
GaMATE24	GaMATE66	244.1667	849.8333	350.8333	1134.167	0.696	0.7493	1.9728	5.2347	0.3769	0.9288	Yes
GaMATE21	GaMATE42	222.1667	764.8333	314.6667	1020.333	0.706	0.7496	2.1276	5.6368	0.3774	0.9419	Yes
GaMATE26	GrMATE46	280.3333	917.6667	390.1667	1223.833	0.7185	0.7498	2.3775	6.293	0.3778	0.9582	Yes
GrMATE26	GrMATE63	247.6667	797.3333	337.8333	1063.167	0.7331	0.75	2.8447	7.3945	0.3847	0.9775	Yes
GaMATE16	GaMATE63	251.3333	829.6667	348.5	1106.5	0.7212	0.7498	2.4444	6.2174	0.3932	0.9618	Yes
GrMATE29	GrMATE43	217.1667	756.8333	315.1667	1010.833	0.6891	0.7487	1.8826	4.7812	0.3937	0.9203	Yes
GaMATE23	GaMATE43	233.3333	814.6667	323.5	1086.5	0.7213	0.7498	2.4468	6.2037	0.3944	0.9619	Yes
GaMATE14	GrMATE62	224	810	330.1667	1082.833	0.6784	0.748	1.7622	4.4594	0.3952	0.907	Yes
GaMATE8	GrMATE52	231.1667	764.8333	331.8333	1021.167	0.6966	0.749	1.9822	4.9501	0.4004	0.9301	Yes
GaMATE22	GaMATE35	231.6667	806.3333	330.5	1076.5	0.701	0.749	2.0455	4.9897	0.41	0.9358	Yes
GaMATE35	GrMATE34	267.8333	907.1667	379.1667	1210.833	0.7064	0.7492	2.1333	5.1405	0.415	0.9428	Yes
GaMATE49	GaMATE63	243.6667	816.3333	352	1091	0.6922	0.7482	1.9228	4.5424	0.4233	0.9251	Yes
GaMATE57	GrMATE27	254.8333	863.1667	357.1667	1151.833	0.7135	0.7494	2.2668	5.3297	0.4253	0.9521	Yes
GaMATE33	GaMATE66	249.3333	843.6667	344.6667	1125.333	0.7234	0.7497	2.5045	5.8776	0.4261	0.9649	Yes
GrMATE16	GrMATE44	254.1667	872.8333	364	1166	0.6983	0.7486	2.0054	4.6971	0.4269	0.9328	Yes
GrMATE24	GrMATE29	215.1667	756.8333	313.6667	1012.333	0.686	0.7476	1.8456	4.3125	0.428	0.9176	Yes
GaMATE64	GrMATE35	262.5	901.5	366.1667	1202.833	0.7169	0.7495	2.3401	5.4561	0.4289	0.9565	Yes
GaMATE40	GrMATE29	216.8333	758.1667	312.6667	1013.333	0.6935	0.7482	1.9393	4.5204	0.429	0.9269	Yes
GaMATE46	GaMATE57	256.6667	863.3333	357.1667	1151.833	0.7186	0.7495	2.3804	5.5309	0.4304	0.9588	Yes
GaMATE48	GrMATE25	71	267	104.5	357.5	0.6794	0.7469	1.7726	4.1053	0.4318	0.9097	Yes
GrMATE30	GrMATE58	273.1667	919.8333	383.3333	1227.667	0.7126	0.7493	2.249	5.1841	0.4338	0.9511	Yes
GaMATE36	GaMATE56	224.1667	739.8333	319.6667	988.3333	0.7013	0.7486	2.05	4.695	0.4366	0.9368	Yes
GaMATE3	GaMATE63	243.1667	818.8333	343.6667	1093.333	0.7076	0.7489	2.1541	4.9164	0.4381	0.9448	Yes
GrMATE11	GrMATE25	238.3333	831.6667	350	1114	0.681	0.7466	1.789	4.0382	0.443	0.9121	Yes
GaMATE68	GrMATE19	241	850	340.8333	1135.167	0.7071	0.7488	2.1457	4.8213	0.4451	0.9443	Yes
GrMATE42	GrMATE68	223.1667	774.8333	315.1667	1034.833	0.7081	0.7488	2.1634	4.7988	0.4508	0.9457	Yes
GrMATE60	GrMATE61	260.6667	892.3333	375.3333	1193.667	0.6945	0.7476	1.9527	4.295	0.4546	0.929	Yes
GrMATE15	GrMATE33	146.8333	538.1667	208	719	0.7059	0.7485	2.1257	4.6576	0.4564	0.9431	Yes
GrMATE17	GrMATE58	258.1667	871.8333	367.6667	1165.333	0.7022	0.7481	2.0644	4.4999	0.4588	0.9386	Yes
GaMATE15	GrMATE58	258.6667	871.3333	368.3333	1164.667	0.7023	0.7481	2.0658	4.4995	0.4591	0.9387	Yes
GrMATE33	GrMATE41	216.8333	791.1667	319	1061	0.6797	0.7457	1.7758	3.8676	0.4591	0.9116	Yes
GaMATE43	GrMATE6	229	811	325.8333	1084.167	0.7028	0.748	2.0745	4.4603	0.4651	0.9395	Yes
GrMATE7	GrMATE53	243.3333	808.6667	336.8333	1079.167	0.7224	0.7493	2.4771	5.2808	0.4691	0.9641	Yes
GrMATE22	GrMATE40	238.1667	836.8333	343.5	1120.5	0.6934	0.7468	1.9374	4.1019	0.4723	0.9284	Yes
GaMATE17	GrMATE50	235	794	326.1667	1059.833	0.7205	0.7492	2.4265	5.1088	0.475	0.9617	Yes
GaMATE10	GaMATE53	225.5	829.5	328	1112	0.6875	0.746	1.8637	3.9166	0.4758	0.9216	Yes
GaMATE2	GrMATE14	251.8333	853.1667	349.1667	1138.833	0.7212	0.7492	2.4458	5.0945	0.4801	0.9627	Yes
GrMATE13	GrMATE33	270.8333	941.1667	380.5	1257.5	0.7118	0.7484	2.2326	4.6328	0.4819	0.951	Yes
GaMATE56	GrMATE8	244.3333	828.6667	357.6667	1112.333	0.6831	0.745	1.813	3.7551	0.4828	0.917	Yes
GaMATE31	GrMATE44	251.5	870.5	363.3333	1166.667	0.6922	0.7461	1.9223	3.9526	0.4863	0.9277	Yes
GrMATE39	GrMATE58	236	856	342.8333	1148.167	0.6884	0.7455	1.8743	3.8431	0.4877	0.9233	Yes
GaMATE61	GrMATE16	247.8333	846.1667	348.1667	1130.833	0.7118	0.7483	2.2334	4.5532	0.4905	0.9513	Yes
GrMATE48	GrMATE60	281	895	381.3333	1193.667	0.7369	0.7498	3.0349	6.1375	0.4945	0.9828	Yes
GaMATE12	GaMATE56	244	859	349.3333	1150.667	0.6985	0.7465	2.0085	4.0306	0.4983	0.9356	Yes
GaMATE34	GrMATE59	228.5	845.5	334	1136	0.6841	0.7443	1.8243	3.6568	0.4989	0.9192	Yes
GaMATE53	GrMATE39	231.8333	856.1667	339.6667	1151.333	0.6825	0.7436	1.8063	3.5764	0.5051	0.9178	Yes
GrMATE23	GrMATE35	233.1667	809.8333	331.6667	1084.333	0.703	0.7468	2.0777	4.1043	0.5062	0.9413	Yes
GaMATE5	GrMATE49	244.8333	850.1667	340.3333	1135.667	0.7194	0.7486	2.3991	4.7158	0.5087	0.961	Yes
>GaMATE1	GrMATE11	231.5	762.5	326.8333	1020.167	0.7083	0.7474	2.1674	4.2562	0.5092	0.9477	Yes
GrMATE6	GrMATE70	243	829	342.8333	1109.167	0.7088	0.7474	2.1762	4.2507	0.512	0.9483	Yes
GrMATE64	GrMATE65	265.3333	861.6667	376.3333	1153.667	0.705	0.7469	2.1109	4.115	0.513	0.944	Yes
GaMATE66	GrMATE51	235.3333	810.6667	335.6667	1086.333	0.7011	0.7462	2.0476	3.972	0.5155	0.9395	Yes
GaMATE11	GaMATE37	242.8333	812.1667	339.3333	1085.667	0.7156	0.7481	2.3119	4.4762	0.5165	0.9566	Yes
GaMATE52	GrMATE50	233.6667	795.3333	323.6667	1062.333	0.7219	0.7487	2.4642	4.7492	0.5189	0.9643	Yes
GaMATE65	GrMATE3	103.3333	386.6667	161.3333	528.6667	0.6405	0.7314	1.4431	2.7727	0.5205	0.8757	Yes
GaMATE58	GrMATE38	235.8333	849.1667	337.1667	1138.833	0.6995	0.7456	2.0229	3.8618	0.5238	0.9381	Yes
GrMATE5	GrMATE29	207	745	314.3333	1011.667	0.6585	0.7364	1.5781	3.008	0.5246	0.8943	Yes
GaMATE38	GrMATE14	174.6667	587.3333	241.5	784.5	0.7233	0.7487	2.5004	4.7524	0.5261	0.9661	Yes
GaMATE59	GrMATE48	284.3333	894.6667	382	1193	0.7443	0.7499	3.6634	6.9611	0.5263	0.9925	Yes
GrMATE41	GrMATE51	217.1667	779.8333	324.6667	1055.333	0.6689	0.7389	1.6682	3.1629	0.5274	0.9052	Yes
GaMATE50	GaMATE51	254.8333	836.1667	350.3333	1116.667	0.7274	0.7488	2.6267	4.8321	0.5436	0.9714	Yes
GrMATE56	GrMATE65	133	446	184	596	0.7228	0.7483	2.4884	4.5769	0.5437	0.9659	Yes
GrMATE19	GrMATE46	257.8333	864.1667	361.3333	1156.667	0.7136	0.7471	2.2683	4.1712	0.5438	0.9551	Yes
GrMATE1	GrMATE67	237.6667	815.3333	341.1667	1095.833	0.6966	0.744	1.9821	3.625	0.5468	0.9363	Yes
GaMATE19	GrMATE17	263.5	841.5	359	1123	0.734	0.7493	2.8848	5.2678	0.5476	0.9795	Yes
GrMATE2	GrMATE51	245.5	811.5	338	1084	0.7263	0.7486	2.5919	4.7214	0.549	0.9702	Yes
GrMATE43	GrMATE64	256.5	870.5	362.8333	1167.167	0.7069	0.7458	2.143	3.8929	0.5505	0.9479	Yes
GrMATE45	GrMATE48	249.6667	810.3333	345	1083	0.7237	0.7482	2.5121	4.5369	0.5537	0.9672	Yes
GrMATE20	GrMATE27	270.6667	927.3333	372.1667	1238.833	0.7273	0.7486	2.6224	4.6883	0.5593	0.9716	Yes
GaMATE30	GrMATE60	274	899	374.6667	1200.333	0.7313	0.749	2.7693	4.9346	0.5612	0.9764	Yes
GaMATE32	GrMATE42	263	856	364.3333	1144.667	0.7219	0.7478	2.4623	4.3792	0.5623	0.9653	Yes
GrMATE32	GrMATE67	234.3333	798.6667	332	1072	0.7058	0.745	2.1239	3.7617	0.5646	0.9474	Yes
GaMATE4	GaMATE45	230.1667	808.8333	338.8333	1095.167	0.6793	0.7385	1.7711	3.1364	0.5647	0.9198	Yes
GrMATE38	GrMATE40	220	835	328	1136	0.6707	0.735	1.6854	2.9358	0.5741	0.9125	Yes
GaMATE6	GrMATE21	253.1667	838.8333	348.6667	1121.333	0.7261	0.7481	2.5846	4.4711	0.5781	0.9706	Yes
GaMATE47	GrMATE44	231.3333	747.6667	313.3333	997.6667	0.7383	0.7494	3.1202	5.3676	0.5813	0.9852	Yes
GrMATE14	GrMATE27	239.6667	844.3333	347.3333	1140.667	0.69	0.7402	1.8945	3.2541	0.5822	0.9322	Yes
GrMATE52	GrMATE59	231.5	788.5	337.1667	1066.833	0.6866	0.7391	1.853	3.1737	0.5839	0.929	Yes
GaMATE60	GrMATE3	109.8333	388.1667	162.3333	527.6667	0.6766	0.7356	1.743	2.9661	0.5876	0.9197	Yes
GrMATE9	GrMATE14	251.1667	852.8333	347	1141	0.7238	0.7474	2.5164	4.2612	0.5905	0.9684	Yes
GaMATE13	GrMATE35	246.3333	841.6667	344.5	1128.5	0.715	0.7458	2.2995	3.8937	0.5906	0.9587	Yes
GaMATE25	GrMATE20	268.1667	890.8333	366.8333	1190.167	0.731	0.7485	2.758	4.6583	0.5921	0.9767	Yes
GaMATE42	GaMATE44	227.6667	756.3333	321.6667	1016.333	0.7078	0.7442	2.1577	3.6439	0.5922	0.9511	Yes
GrMATE25	GrMATE66	252.1667	841.8333	338.3333	1122.667	0.7453	0.7499	3.8076	6.3957	0.5953	0.994	Yes
GaMATE39	GrMATE44	255.1667	884.8333	357.6667	1187.333	0.7134	0.7452	2.2654	3.7929	0.5973	0.9573	Yes
GaMATE18	GrMATE63	243.5	790.5	340.1667	1060.833	0.7158	0.7452	2.3164	3.7837	0.6122	0.9606	Yes
GaMATE51	GrMATE65	271.8333	885.1667	376.8333	1186.167	0.7214	0.7462	2.449	3.972	0.6166	0.9667	Yes
GrMATE47	GrMATE54	243.1667	806.8333	337.5	1081.5	0.7205	0.746	2.4266	3.9313	0.6173	0.9658	Yes
GrMATE61	GrMATE66	235.6667	827.3333	340.3333	1120.667	0.6925	0.7383	1.9257	3.1172	0.6178	0.938	Yes
GrMATE35	GrMATE36	244.6667	839.3333	344.1667	1128.833	0.7109	0.7435	2.2154	3.5659	0.6213	0.9561	Yes
GrMATE27	GrMATE50	222	778	325.5	1060.5	0.682	0.7336	1.8007	2.8678	0.6279	0.9297	Yes
GrMATE51	GrMATE70	242.6667	807.3333	337.8333	1084.167	0.7183	0.7447	2.3729	3.7083	0.6399	0.9646	Yes
GrMATE67	GrMATE69	223.1667	805.8333	331.1667	1105.833	0.6739	0.7287	1.7158	2.6714	0.6423	0.9248	Yes
GrMATE66	GrMATE67	224.1667	806.8333	330	1107	0.6793	0.7288	1.7711	2.6762	0.6618	0.932	Yes
GaMATE54	GaMATE60	235.6667	761.3333	323.8333	1020.167	0.7277	0.7463	2.638	3.9804	0.6627	0.9752	Yes
GrMATE63	GrMATE67	233.8333	790.1667	331.8333	1069.167	0.7047	0.739	2.1046	3.17	0.6639	0.9535	Yes
GaMATE41	GaMATE49	245.3333	811.6667	346.5	1096.5	0.708	0.7402	2.1624	3.2559	0.6641	0.9565	Yes
GrMATE57	GrMATE63	245.6667	790.3333	339.1667	1061.833	0.7243	0.7443	2.5309	3.661	0.6913	0.9731	Yes
GrMATE65	GrMATE66	241	817	346.5	1114.5	0.6955	0.7331	1.9668	2.843	0.6918	0.9488	Yes
GrMATE49	GrMATE50	232.3333	790.6667	322.1667	1063.833	0.7212	0.7432	2.4437	3.53	0.6923	0.9703	Yes
GrMATE37	GrMATE57	233	795	329.5	1077.5	0.7071	0.7378	2.1465	3.0901	0.6946	0.9584	Yes
GrMATE58	GrMATE60	280.1667	895.8333	378.3333	1196.667	0.7405	0.7486	3.2789	4.7166	0.6952	0.9892	Yes
GrMATE36	GrMATE41	216.3333	766.6667	319.3333	1060.667	0.6775	0.7228	1.7519	2.4881	0.7041	0.9372	Yes
GrMATE50	GrMATE65	229.1667	766.8333	332.5	1053.5	0.6892	0.7279	1.8846	2.6431	0.7131	0.9469	Yes
GrMATE40	GrMATE50	218.6667	769.3333	321.6667	1064.333	0.6798	0.7228	1.7765	2.4885	0.7139	0.9405	Yes
GrMATE59	GrMATE70	239.6667	819.3333	342	1119	0.7008	0.7322	2.0428	2.8057	0.7281	0.9571	Yes
GrMATE68	GrMATE69	208.1667	745.8333	309.5	1040.5	0.6726	0.7168	1.7032	2.3382	0.7284	0.9383	Yes
GaMATE55	GaMATE66	239.8333	829.1667	342.5	1133.5	0.7002	0.7315	2.0347	2.7771	0.7327	0.9573	Yes
GrMATE62	GrMATE68	214.1667	754.8333	309.8333	1040.167	0.6912	0.7257	1.9099	2.5717	0.7426	0.9525	Yes
GrMATE4	GrMATE13	252.6667	826.3333	341.1667	1104.833	0.7406	0.7479	3.2842	4.4179	0.7434	0.9902	Yes
GrMATE10	GrMATE67	238.1667	802.8333	339	1098	0.7026	0.7312	2.0704	2.7638	0.7491	0.9609	Yes
GrMATE54	GrMATE57	233.3333	780.6667	334	1073	0.6986	0.7276	2.0104	2.6318	0.7639	0.9602	Yes
GrMATE18	GrMATE45	254.6667	813.3333	341.8333	1086.167	0.745	0.7488	3.7583	4.8351	0.7773	0.9949	Yes
GrMATE55	GrMATE57	232.3333	774.6667	335.1667	1071.833	0.6932	0.7227	1.9352	2.4862	0.7784	0.9591	Yes
GrMATE31	GrMATE63	244.1667	773.8333	342.8333	1058.167	0.7122	0.7313	2.2409	2.7685	0.8094	0.9739	Yes
GrMATE53	GrMATE57	237.5	777.5	335.5	1071.5	0.7079	0.7256	2.16	2.5697	0.8406	0.9756	Yes
GrMATE28	GrMATE47	250	823	347.1667	1128.833	0.7201	0.7291	2.417	2.6842	0.9005	0.9877	Yes
GrMATE69	GrMATE70	247.5	813.5	341	1120	0.7258	0.7263	2.5755	2.5922	0.9936	0.9993	Yes

Sn, The number of observed non-synonymous substitutions; s, number of synonymous sites; n, number of nonsynonymous sites; S, number of synonymous substitutions; N, number of nonsynonymous substitutions; ps, probability of rejecting the null hypothesis; pn, the proportion of observed non-synonymous substitutions; ds, synonymous substitution rate; dn, nonsynonymous substitution; ds/dn, selective strength of sequence; ps/pn, the ratio of observed synonymous substitution to the proportion of observed non-synonymous substitutions.

### Promoter cis-element analysis

**Figure 5:**
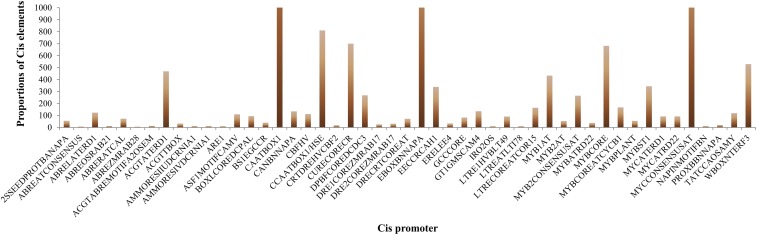
Average number of *cis*-promoter elements in the regions of *G. raimondii* and *G. arboreum MATE* genes. The *cis*-promoters were analyzed in the 1 kb up/down stream promoter regions of translation start site, using the PLACE database.

Promoter sequences 2 kb upstream and downstream of the translation start and stop site of all *MATE* genes were obtained from the Cotton Genome Project. Transcriptional response elements of *MATE* genes promoters were predicted by the PLACE database (http://www.dna.affrc.go.jp/PLACE/signalscan.html) (Higo *et al.* 1999). In order to determine the *cis*-acting regulator elements, we queried a section of the sequence of each gene, but only the start and end codons were used for the selection of *cis*-promoter elements. Using the PLACE database, we identified several putative-stress, *cis*-acting elements in both *GrMATE* and *GaMATE* genes (Figure 5 and Table S5).

We detected commonly known *cis*-promoter elements associated with stress in a number of genes: HSE/CCAATBOX1 (heat stress-responsive element), LTR/LTRE1HVBLT49/LTREATLTI78 (low temperature-responsive element), BOXLCOREDCPAL/MYBST1 (MYB-binding site), WBOXNTERF3/WUN (wound-responsive element), CURECORECR (copper-responsive element), ABRELATERRD1 (early response to dehydration), ABREZMRAB28 (cold/freezing tolerance), ABRERACAL (Ca^2+^ response), and ABRE (ABA-responsive element).

In general, the total stress and/or hormonal *cis*-acting elements were 18, and close to half were majorly responsible for stress-related activities. In most of the *MATE* genes, we detected more than one *cis*-acting element, and so our results agree with previous findings that heat stress transcription factors and heat-shock element (HSE) were found to be consistently conserved in the regulatory region of heat-induced genes (Nover *et al.* 1996; Larkindale and Vierling 2007). Among the stress-related *cis*-elements detected in this study were HSE/CAATBOX1 (CAAT) and EBOXBNNAPA (CANNTG) repeats, whereas ABREZMRAB28 (CCACGTGG) was the least detected but was common among the various *MATE* genes. The HSE is a stress-responsive element that is important in the ABA signaling pathway, initiating plant response to water deficit and high-salinity stress factors (Narusaka *et al.* 2003). The detection of these promoter elements being associated with cotton *MATE* genes points to their vital role in enhancing drought and salt stress tolerance. A significantly high number of *GrMATE* and *GaMATE* genes were found to contain long terminal repeat (LTR) element, which is a *cis*-element responsive to low-temperature stress, the same as that identified in barley (Brown *et al.* 2001).

High proportions of *GaMATE* and *GrMATE* genes were found to contain BOXLCOREDCPAL/MYBST1, a binding site for MYB, which is known to be involved in drought stress induction in plants (Shukla *et al.* 2015). TC-rich repeat elements were detected in 52 *GrMATE* and 45 *GaMATE* genes. TC-rich repeat is a promoter element that has been found to be involved in defense and stress responsiveness in dehydrating responsive element binding (*DREB*) gene of *Arabidopsis* (Sazegari *et al.* 2015). Furthermore, ABRE, which is associated with the ABA-dependent signaling pathway, was found to be contained in a number of *GrMATE* and *GaMATE* genes. ABRE is mainly vital for ABA signaling, and enhances plant response to drought and salt stress (Shinozaki and Yamaguchi-Shinozaki 2000).

### GrMATE and GrMATE genes functional determination by GO annotation

**Figure 6:**
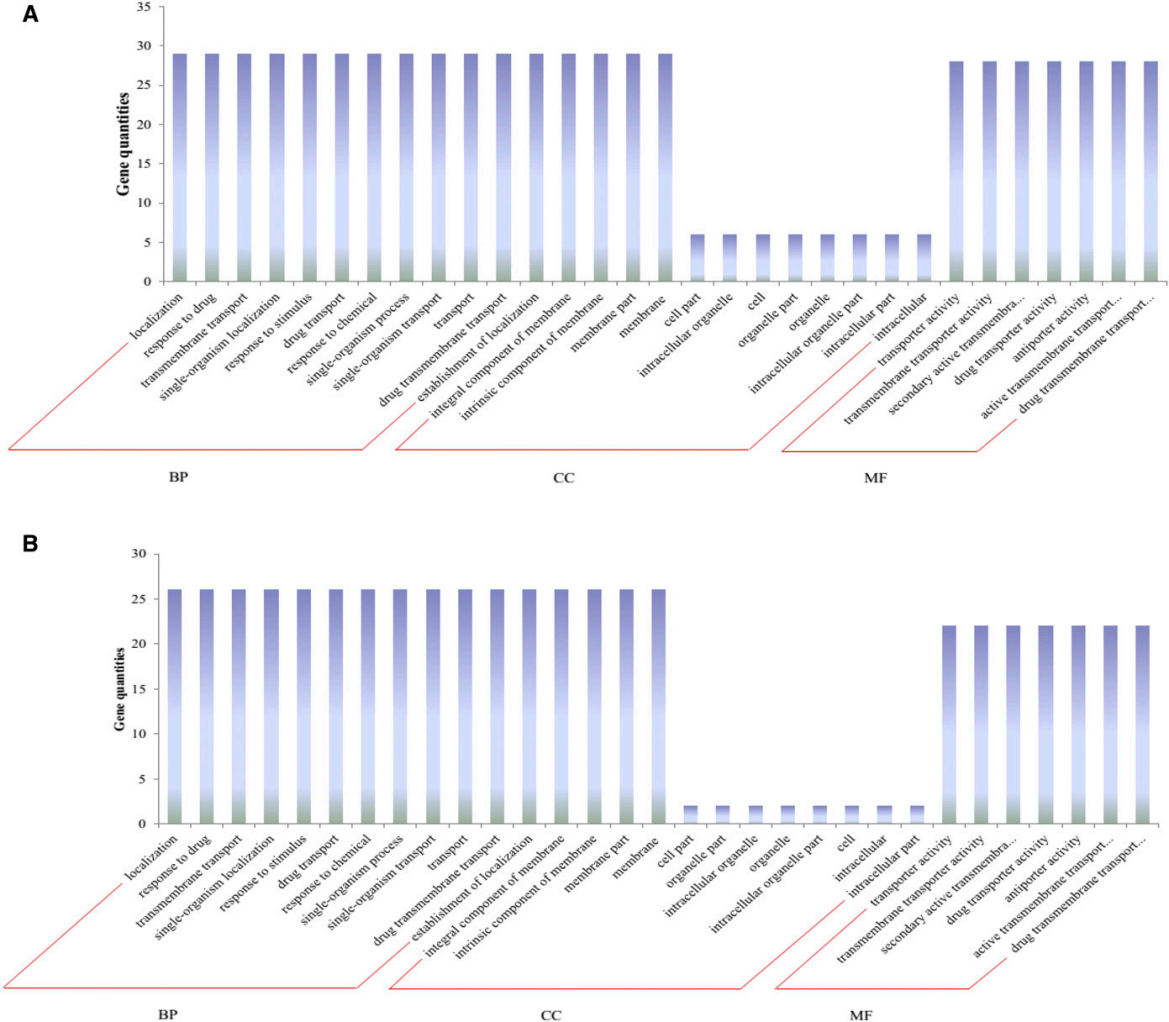
Gene ontology (GO) annotation results for diploid cotton *MATE* genes. GO analysis of (A) upregulated and (B) downregulated MATE protein sequences predicted for their involvement in biological processes, molecular functions, and cellular processes.

The BP, MF, and CC of diploid cotton *MATE* genes were examined as per the GO database. Blast2GO v4.0 was used to carry out the analysis (Figure 6 and Table S6). The results showed that 135 *MATE* genes were putatively involved in arrange of biological, cellular, and molecular processes within the plant. In all GO annotations, all 135 *MATE* genes were involved in the three GO functional annotation. In specificity, for CC, the genes were found to function in the membrane, membrane part, cell part, organelle part, organelle, micromolecular complex, and the cell. In MF, the genes were found to be involved in processes such as transporter activity, transmembrane transporter activity, secondary active transmembrane, drug transporter activity, antiporter activity, active transmembrane transporter activity, and finally, drug transmembrane transporter activity. In BP, functions such as response to stimulus, regulation of biological process, developmental process, biological regulation, multicellular organismal process, and single organism response were detected (Figure 6).

In all GO function annotations, different functions were noted with various GO annotation exhibiting diverse roles. In relation to the MF, the following GO functional annotation was noted for salt, Cd and drought stress: antiporter activity (GO: 0015297), drug transmembrane transporter activity (GO: 0015238), motor activity (GO: 0003774), and ATP binding (GO: 0005524). Higher plants are known to have a multitude of Multiple Drug Resistance (MDR) transporter homologs, in which MATE forms one of the larger components. MDR transporters have a primary contribution to cellular detoxification processes in plants, which mainly occurs by the extrusion of toxic compounds from the cell or their sequestration in the central vacuole (Park *et al.* 2012; Remy and Duque 2014; Shoji 2014). The ATP-binding role of the *MATE* genes enables the plants to tolerate Cd stress through complexing Cd ions with metal-chelating peptides such as phytochelatins, metallothionein, and glutathione, making the Cd ions form complexes that are nontoxic and easily eliminated from the cells (Howden *et al.* 1995; Cobbett *et al.* 1998).

In BP, the following functional annotations were found to cut across all the three stress levels: drug transmembrane transport (GO: 0006855), iron ion homeostasis (GO: 0055072), and transmembrane transport (GO: 0055085); under salt stress, two unique functions were observed: cellular response to carbon dioxide (GO: 0071244) and regulation of stomatal opening (GO: 1902456). Because of global warming, CO_2_ levels have increased tremendously, posing a challenge to plant survival despite it being a raw material for plants in the photosynthesis process. Increased levels of CO_2_ do lead to elevation of cytoplasmic bicarbonate concentration, which in turn, activates anion channels in guard cells required for stomatal closing, hindering the normal process of photosynthesis (Xue *et al.* 2011). Recent studies have shown that the MATE transporter-like protein RHC1 functions as a bicarbonate sensor and initiates various mechanisms for its regulations in plant cells (Tian *et al.* 2015).

In CC, integral component of membrane (GO:0016021), myosin complex (GO:0016459), Golgi transport complex (GO:0017119), vacuolar membrane (GO:0005774), and membrane (GO:0016020) were found across all three stress factors, which gave a clear indication that GrMATE and GaMATE have a functional role in the maintaining of the cellular membrane structure integrity. Plasma membrane (GO: 0005886) and chloroplast (GO: 0009507) were detected under salt and drought stress, respectively.

In all of the MATE groups, MF, BP, and CC were noted except in one single *MATE* gene, GaMATE48 (*Cotton_A_25608*), in which none of the GO functions were detected. The various GO functional annotations have also been observed for various stress-related genes such as *LEA* genes (Magwanga *et al.* 2018).

### Analysis of tertiary protein structure of diploid cotton MATE proteins

**Figure 7:**
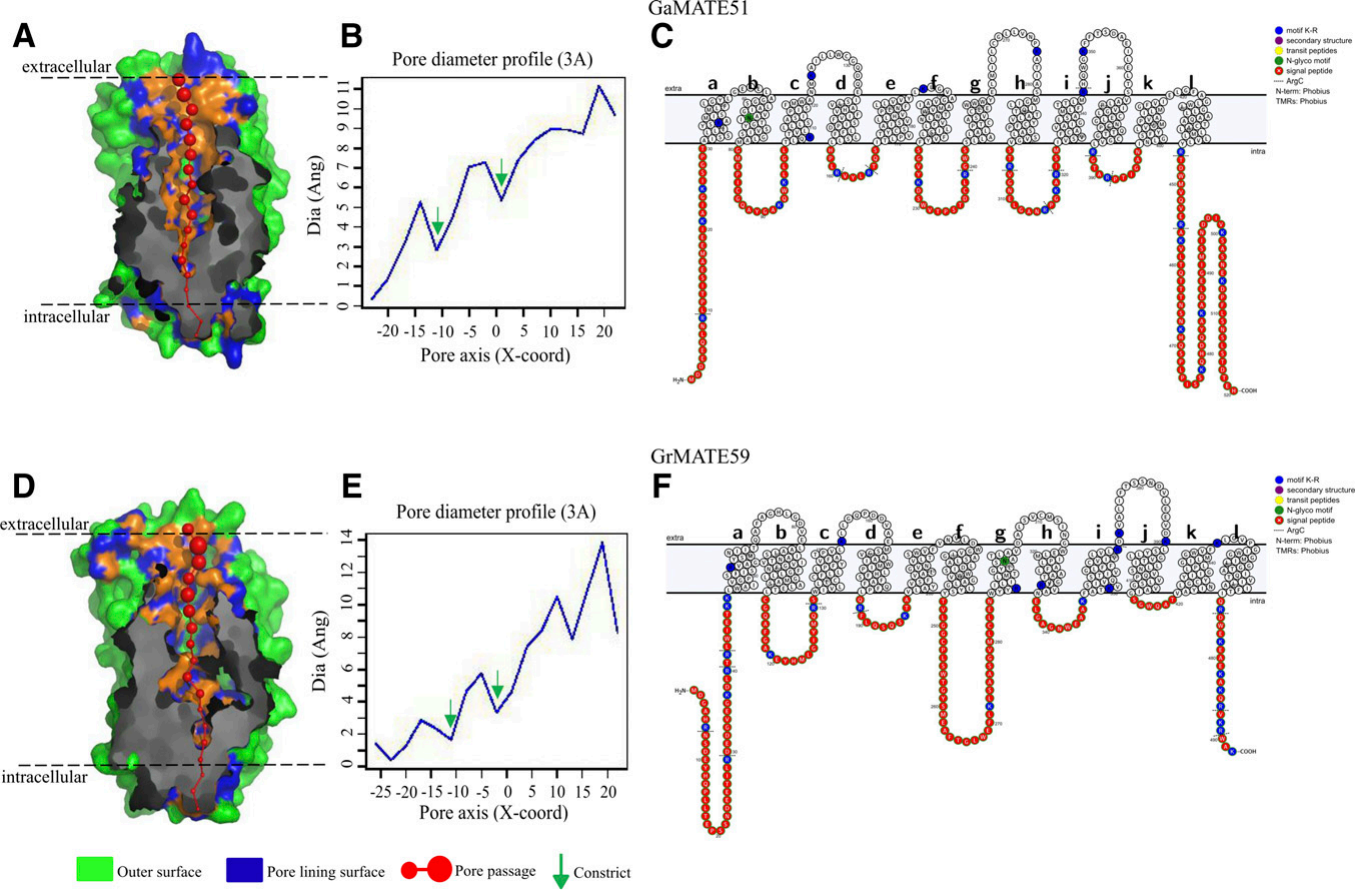
Pore morphology, dimensions, and protein topology of *G. arboreum and G. raimondii* MATE proteins. (A and D) Protein tertiary structures showing pore morphology of MATE family members. (B and E) Graphs showing pore dimensions obtained from PoreWalker software. (C and F) Topology of two examples of two MATE proteins.

The protein secondary structure of all 70 GrMATE and 68 GaMATE proteins were predicted to form hourglass-like structure with 3–12 TMs, and similar secondary structures have been identified among membrane proteins such as aquaporins (Maurel *et al.* 2008). Pore structure and 3D geometry of a channel of all MATE family members were obtained with PoreWalker software, which identified a pore that longitudinally traversed through the extracellular to intracellular opening of the protein. The pore morphology clearly showed conservation of pore size and two constraints that were known to act as selectivity barriers in the pore (Figure 7). Although PoreWalker analysis does not provide information about solute interaction, the information of pore morphology obtained aids in predicting solute permeability (Vogel 2000). Conservation of pore size and similar constraint in all the MATEs showed that the genes could be involved in the exclusion role of substances from the cell. The results obtained, were further validated by using an online tool for structure visualization, Protter (http://wlab.ethz.ch/protter/). The MATE proteins were found to be membrane proteins, which transverse the intra and extracellular region of the membranes (Figure 7 and Table S3). The orientation of these proteins in the cell membrane could be facilitating the removal of solutes and other harmful substances in order to reduce the injuries caused during stress conditions. MATE proteins are membrane proteins and possess pore forming amino acids, which makes them substrate-specific; similar attributes have been reported among aquaporins, which are known to be substrate-specific because of the size of the amino acids that form pores (Fu 2000; Lee *et al.* 2005; Törnroth-Horsefield *et al.* 2006).

### Transcriptional responses of cotton MATE genes under salt, drought and Cd treatment

**Figure 8:**
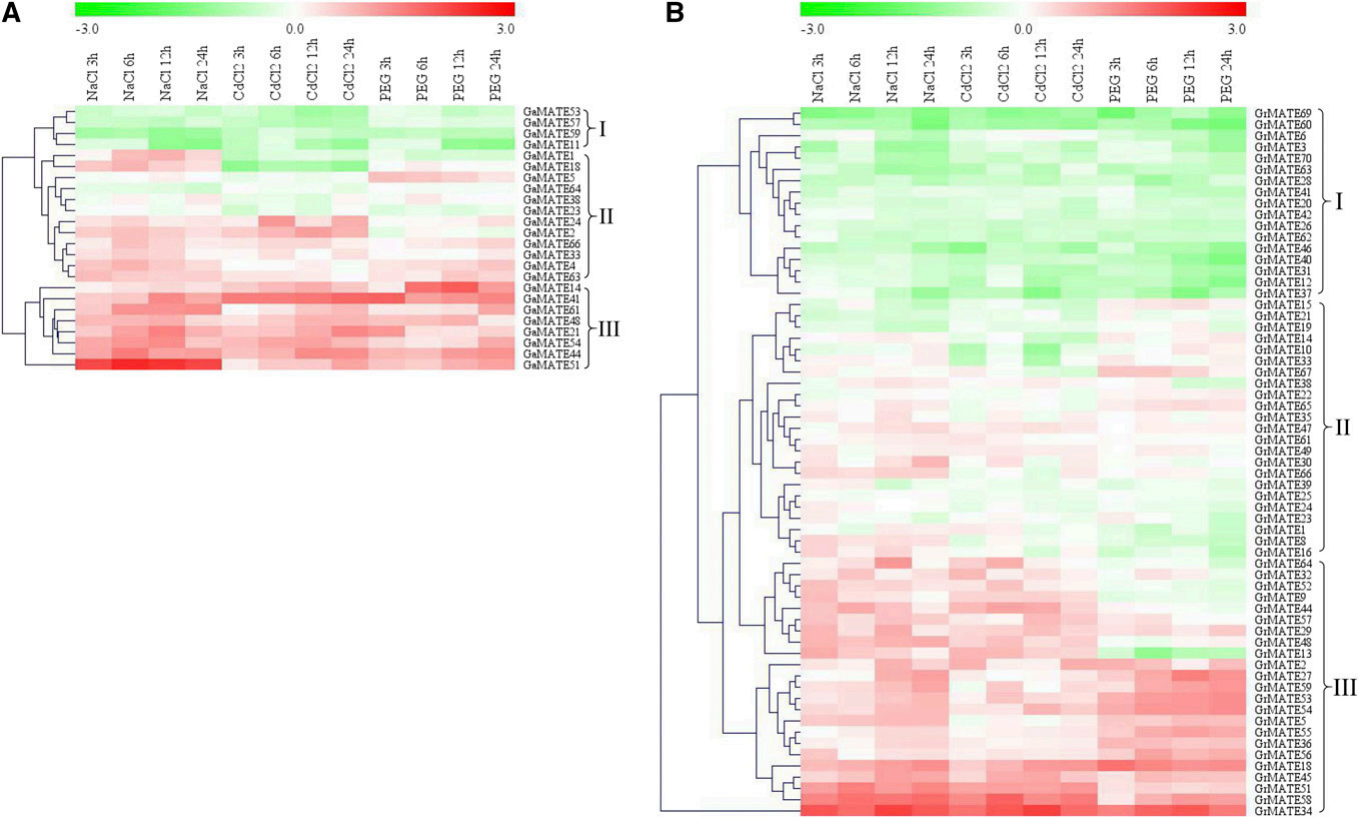
Differential expression of diploid cotton *MATE* genes under drought, salt, and Cd stress. The heat map was visualized using the MeV_4_9_0 program. Red and green indicate high and low levels of expression, respectively. (A) Heat map showing 24 *GaMATE* genes. (B) Heat map for the 63 genes of *G. raimondii* (GrMATEs).

There is an increased body of evidence showing that the *MATE* genes are significantly important in conferring tolerance to various abiotic stress factors. Expression profiling of the *GaMATE* and *GrMATE* genes was done on the root tissues of *G. arboreum* and *G. raimondii* cotton plant in order to examine their expression levels in the root tissues under drought, salt and Cd stress. Previous studies showed that inhibition of root elongation is the most sensitive parameter of Cd toxicity (Guo and Marschner 1995). In carrying out the RNA expression validation under salt, drought and cadmium stress conditions, we used 24 *GaMATE* and 63 *GrMATE* genes. The selection of the genes for qRT-PCR analysis was based on the gene structure and phylogenetic tree analysis, with more emphasis on *G. raimondii* of the D genome in which over 89% of the *GrMATE* genes were profiled. In *GaMATE* genes, the expression patterns were clustered into three groups. Group I had four genes, *GaMATE53*, *GaMATE57*, *GaMATE59*, and *GaMATE11*, and all were downregulated. GaMATE57 and GaMATE59 are members of subfamily M2, while GaMATE53 and GaMATE11 are members of subfamily M1. The second group had 12 *GaMATE* genes, all of which exhibited differential expression across the three stress factors, salt, drought and Cd stresses. Only one gene was a member of the M3 subfamily, GaMATE66 (*Cotton_A_00702*), and was upregulated in salt, drought and Cd stress levels while GaMATE23 and GaMATE38, both members of the M1 subfamily, were all downregulated. Group two genes were significantly upregulated under salt stress but exhibited differential expression under drought and Cd stress conditions. Among the group two members, two genes exhibited unique expression patterns, GaMATE18 and GaMATE1, both members of M2 subfamily. Both genes were highly upregulated under salt stress but were downregulated under drought and Cd stress conditions. The third group had eight genes, all of which were significantly upregulated in all three stress levels; GaMATE41, GaMATE44, GaMATE61, GaMATE14, GaMATE21, and GaMATE48 were members of the subfamily M1. The subfamily M1 gene members showed more upregulation compared to M2 and M3 subfamilies, an indication of a larger role for the members of subfamily M1 in enhancing salt, Cd and drought stress tolerance in cotton (Figure 8A).

The D genome is known to harbor vital genes more than the A genome, and therefore we also analyzed the expression profile of 63 *MATE* genes of *G. raimondii* under salt, drought and Cd stress factors. The expression nature of the *GrMATE* genes in three levels of stress showed differential expressions, and not all the genes were upregulated across the three stress levels. Out of the total genes, eight *GrMATE* genes [*GrMATE22* (M1), *GrMATE23* (M3), *GrMATE24* (M3), *GrMATE25* (M3), *GrMATE39* (M1), *GrMATE49* (M1), *GrMATE61* (M1) and *GrMATE35* (M2)] were neither upregulated nor downregulated in all three stress levels despite the stress exposure variation from 0 to 24 hr. This implied that these genes do not have any functional role in the root tissues but could have a role in other tissues not analyzed in this research. The expression profile of the *GrMATE* genes were also clustered into three distinct groups: cluster 1 (17 genes), cluster 2 (23 genes), and cluster 3 (23 genes). More than 75% of the genes in cluster 3 were highly upregulated across the three treatments. Significantly, *GrMATE34* (M2), *GrMATE58* (M2), and *GrMATE18* (M1) exhibited the highest levels of upregulation and could be the key *MATE* genes, with a profound role in salt, drought and Cd stress tolerance in cotton (Figure 8B).

## Discussion

MATE proteins are members of secondary active transporters, with wide distribution in all living organisms. Cotton is an important crop and the chief source of raw materials to the textile industry, and the completion of *G. raimondii* (D genome) and *G. arboreum* (A genome) genome sequencing provided an excellent opportunity to carry out genome-wide characterization of the *MATE* gene family in two diploid cottons. In this study, we identified 70 and 68 *MATE* genes in *G. raimondii* of genome D and *G. arboreum* of genome A, respectively. The number of *MATE* genes for the two diploid cottons were relatively closer to that of *Arabidopsis*, with 58 genes (Li *et al.* 2002), even though the genome size of *Arabidopsis* is much smaller compared to that of the two diploid cotton. *Arabidopsis* evolved through polyploidization, and at least four folds of whole-genome duplication events have been recorded in the evolution history of the *Arabidopsis* plant (Vision *et al.* 2000).

Based on phylogenetic tree analysis, *MATE* genes were basically grouped in to three subfamilies, and the intron-exon structures were subfamily-specific, an indication that the cotton *MATE* genes are considerably conserved and are functionally diversified. The exon-intron plays a greater role in the divergence of gene structure, and in turn, their functions within the organism (Fan *et al.* 2014). Introns have been found to alter the activities of genes, and the presence of introns in a genome is believed to impose substantial burden on the host. The excision of spliceosome introns requires a spliceosome, which is among the largest molecular complexes of the cell, comprising five snRNAs and >150 proteins (Wahl *et al.* 2009). Interestingly, the majority of gene members of subfamily M2 for *G. arboreum* and *G. raimondii* were intronless. The lack of introns among subfamily M2 indicates that their gene expansion could possibly be independent of the other gene subfamilies, M1 and M3. The expansion of the *MATE* genes in cotton could be governed by the loss or gain of introns, and the same was observed for *MATE* genes in maize (Zhu *et al.* 2016).

Evolution and expansion of a number of functional genes in living organisms have been found to occur through gene duplication (Taylor and Raes 2004). In the analysis of the evolution pattern of the cotton *MATE* genes, segmental gene duplication was found to be the main driving force as opposed to tandem gene duplication. In the evolution and expression profiling of *MATE* genes in soya beans, more genes were found to have undergone segmental gene duplication, with 60.68% compared to 21.37% tandemly duplicated genes (Liu *et al.* 2016). A unique observation was made, in which ds/dn ratio was <1 in all the duplicated gene pairs, ranging from 0.235 to 0.9936. The ds/dn value is an important tool in investigating the type of selection pressure which acted on the protein coding genes. When the ds/dn ratio is <1, this signifies that the evolution of the proteins encoding the *MATE* genes occurred under beneficial selection; if the ds/dn ratio is >1, then the selection pressure occurred under purifying selection; and when the ratio is 1, then the selection pressure was neutral (Magwanga *et al.* 2018). The results indicate that the cotton *MATE* genes have largely undergone purifying selection.

The determination of the subcellular localization of GaMATE and GrMATE transporters is important for the deeper understanding of their critical roles within the plant cell. The majority of MATE proteins characterized in plants so far have been found to be embedded either in the plasma membranes or vacuolar membranes, which are the primary sites for the sequestration and iron uptake (Jeong and Lou Guerinot 2009). When the MATE transporters are localized within the plasma membrane, they enhance the exclusion of substances from the cells in exchange for hydrogen ion influx, but when localized within the vacuolar membrane, they work as uptake transporters because of variation in pH between the cytosol and vacuolar lumen; the cytosolic pH range is (7.2–7.5), which is higher than that of the vacuolar lumen, with a pH of 5.5 (Morita *et al.* 2009). In this research we found that the majority of GaMATE and GrMATE transporters were predicted to be compartmentalized within the plasma membrane, 51 and 54 genes, respectively. The high number of the MATE transporters within the plasma membrane, vacuole, chloroplast, and cytoplasm explains their vital role of compartmentalization of the substrates, which are presumed to be toxic to the plants. Vacuolar compartmentalization of the toxic substances could possibly improve the efficiency of their production and eliminate cell damage (Roytrakul and Verpoorte 2007).

*MATE* genes are known to be involved in the exclusions of toxins, and this function was further evident when the majority of GaMATEs and GrMATEs were found to be involved in secretory pathways. Transport through the secretory pathway begins with translocation of the protein to the endoplasmic reticulum (ER), where the protein is glycosylated, phosphorylation occurs, and disulfide bridges are formed (Porter *et al.* 2015). After passing a sophisticated quality control mechanism, the cargo is transported in vesicles from the ER to the Golgi apparatus (Lin *et al.* 1999; Spang 2013; Kurokawa *et al.* 2014). Interestingly, some of the MATE transporters were predicted to be localized in mitochondrion, extracellular structures, and nuclei, although in low numbers. Similarly, *ZRZ*, a large gene family encoding MATE transporters, have been reported to be localized in mitochondria, indicating that the *ZRZ* genes could be involved in a complex network of communication whereby a leaf-borne signal is responsible for organ initiation (Burko *et al.* 2011). In the recent past, bush and chlorotic dwarf1 *(BCD1)* was found to be localized in the Golgi apparatus and was associated with the role of excretion of excess iron produced in the chlorotic cells in the senescing leaf cells under drought stress conditions (Seo *et al.* 2012).

The *cis*-acting regulatory elements in the promoter regions play an important part in plant response to stresses (Yamaguchi-Shinozaki and Shinozaki 2005). Using the PlantCARE database, we identified 18 putative stress or hormone-responsive *cis*-acting elements in the 1 kb upstream of *GrMATE* and *GaMATE* genes. In relation to stress and stress-related factors, seven putative stress *cis*-acting elements were found in both *GrMATE* and *GaMATE* genes, except for two genes, *GaMATE8 (Cotton_A_08859)* and *GrMATE42 (Gorai.008G097100)*. The *cis*-promoters associated with stress were HSE, LTR (low-temperature responsive element), MBS (MYB binding site), TC-rich repeats (defense and stress responsive element), WUN motif (wound-responsive element), O_2_ site (zinc stress), and ABRE (ABA-responsive element). ABA is synthesized *de novo* mainly for its response to drought and high-salinity stress (Nambara and Marion-Poll 2005). TCA-element is known to be responsible for the mediation of the salicylic acid signaling pathway, in addition to the response to various abiotic stress factors (Mou *et al.* 2013). The detection of these stress-responsive *cis*-elements gave an indication that the proteins encoding the *GrMATE* and *GaMATE* genes have a functional role in salt and drought stress tolerance in the cotton plant.

GO analysis provides the basic possible functions of the genes. It has been used extensively in determining various functions of genes in plants and animals. GO analysis provided three basic fundamental classifications of genes in relation to the part of the cell they function in, namely BP, MF and CC (Ashburner *et al.* 2000). Drought and salt stress are synchronized within the plasma membrane and thus affect the osmotic balance of the cell. In general, the three GO terms highlighted the primary functions of the *MATE* genes to be detoxification and facilitation of the removal of toxins from the plant tissues, thereby maintaining the normal functioning of the cell and that of the cell membrane integrity of various membranous-bound organelles within the cell.

Gene expression analysis is a valuable tool in providing fundamental information on the possible functions of the genes under study. In order to analyze the expression profile of the various cotton *MATE* genes, we carried out the transcriptome expression profiling to determine the transcription expression levels in the root tissues under drought, salt and Cd stress at seedling stage. One hundred and thirty eight genes were investigated and were found to exhibit differential and temporal expression patterns, possibly because of differences in transport substrates or the complex and widespread accumulation of Cd compounds in *G. raimondii* and *G. arboreum*. Six cotton *MATE* genes, *GrMATE2 (Gorai.001G084200)*, *GaMATE4 (Cotton_A_12208)*, *GrMATE3 (Gorai.001G084300)*, *GaMATE3 (Cotton_A_12209)*, *GrMATE41 (Gorai.008G58000)*, and *GaMATE43 (Cotton_A_11428)*, were found to be orthologous to various *MATE* genes of *Arabidopsis*, such as *Ath18*, *Ath19*, *Ath20*, *Ath21*, *Ath16*, *Ath17*, *Ath28*, *Ath31*, and *Ath1*. Of the *MATE* genes from *Arabidopsis*, *Ath19* has been widely investigated and found to be homologous to *AtDTX1*, which has been found to confer Cd stress tolerance in *Arabidopsis* (Li *et al.* 2002), *GrMATE2 (Gorai.001G084200)* exhibited similar expression patterns as *MtMATE2*, which has been found to be strongly expressed in roots (Zhao and Dixon 2009). The flavonoid glycosides have high accumulation in roots, and MtMATE2 mainly transports flavonoids, implying that GrMATE2 *(Gorai.001G084200)* could play a similar role, thus aiding plant adaptability to drought, Cd, and salt stress conditions. *GrMATE26 (Gorai.006G008600)*, *GaMATE58 (Cotton_A_14741)*, *GrMATE42 (Gorai.008G097100)*, and *GaMATE42 (Cotton_A_35442)* were orthologous to *AtTT12* and *MtMATE1*, and are mainly transcribed during the initial stages of silique and young pod development after fertilization in the developing seeds (Diener *et al.* 2001).

*GrMATE54 (Gorai.009G381900)*, *GrMATE53 (Gorai.009G381600)*, and *GaMATE21 (Cotton_A_16784)* were upregulated in the root tissues under salt, Cd and drought stress, and these three genes were orthologous to *DTX19*/*ALF5*. *ALF5* has been found to play a significant role in the vacuolar sequestration and cellular efflux of toxins known to cause plant growth inhibition (Diener *et al.* 2001). *GrMATE34 (Gorai.007G010300)* and *GaMATE54 (Cotton_A_07545)* were significantly upregulated in all three stress levels, and these two genes are ortholog to *DTX50*. The *DTX* carriers are a subfamily of the MATE proteins in *A. thaliana*, and *AtDTX50* mainly functions as an ABA efflux transporter (Diener *et al.* 2001). ABA plays significant roles in various aspects of plant growth and development, including seed germination, senescence, and responses to abiotic stresses (Zhang *et al.* 2006; Fujii *et al.* 2007; Xu *et al.* 2013). These results augment our finding, and clearly elucidate the role of *GaMATE* and *GrMATE* genes in aiding plant survival under drought, Cd and salt stress conditions.In summary, all of our analyses, including bioinformatics and validation by qRT-PCR, clearly indicate that *GrMATE* and *GaMATE* genes do have a putative role in abating the effects of salt, drought and Cd stress in diploid cotton. These genes would provide a much needed molecular approach in improving cotton plants to ever-changing environmental conditions. This study not only shows the functions of *MATE* genes, but also provides a solid foundation for future studies to build upon, such as investigating the transformation and introgression of these genes into the current elite upland cotton.
